# Changes in Blood B Cell-Activating Factor (BAFF) Levels in Multiple Sclerosis: A Sign of Treatment Outcome

**DOI:** 10.1371/journal.pone.0143393

**Published:** 2015-11-23

**Authors:** Karin Kannel, Kristi Alnek, Liina Vahter, Katrin Gross-Paju, Raivo Uibo, Kalle V. Kisand

**Affiliations:** 1 West-Tallinn Central Hospital MS Centre, Tallinn, Estonia; 2 Department of Immunology, Institute of Biomedicine and Translational Medicine, Tartu University, Tartu, Estonia; 3 Institute of Clinical Medicine, Tallinn University of Technology, Tallinn, Estonia; 4 Institute of Psychology, Tallinn University, Tallinn, Estonia; LMU Munich, GERMANY

## Abstract

Multiple sclerosis (MS) is mediated primarily by autoreactive T cells. However, evidence suggesting the involvement of humoral immunity in brain diseases has increased interest in the role of B cells and their products during MS pathogenesis. The major survival factor for B cells, BAFF has been shown to play a role in several autoimmune conditions. Elevated BAFF levels have been reported in MS animal model and during MS relapse in patients. Moreover, disease-modifying treatments (DMT) reportedly influence blood BAFF levels in MS patients, but the significance of these changes remains unclear. The present study addresses how blood BAFF levels are associated with the clinical course of relapsing-remitting MS and the effectiveness of DMT and short-term steroid treatment. During a prospective longitudinal follow-up of 2.3 years, BAFF was measured in the blood of 170 MS patients in the stable phase and within 186 relapses. BAFF levels were significantly higher in MS patients compared to healthy controls. However, stable MS patients without relapses exhibited significantly higher BAFF levels than relapsing patients. Treatment with interferon-β and immunosuppressants raised BAFF blood levels. Interestingly, a similar effect was not seen in patients treated with glatiramer acetate. Short-term treatment with high doses of intravenous methylprednisolone did not significantly alter plasma BAFF levels in 65% of relapsing-remitting MS patients. BAFF were correlated weakly but significantly with monocyte and basophil counts, but not with other blood cell types (neutrophils, lymphocytes, or eosinophils) or inflammatory biomarkers. To our knowledge, this is the first report demonstrating that higher blood BAFF levels may reflect a more stable and effective MS treatment outcome. These results challenge hypotheses suggesting that elevated blood BAFF levels are associated with more severe disease presentation and could explain the recent failure of pharmaceutical trials targeting BAFF with soluble receptor for MS treatment.

## Introduction

Multiple sclerosis (MS) is a progressive autoimmune disease of the CNS. The initial phase of MS is characterized by a relapsing-remitting course in 85% of patients [1]. Specific clinical features of MS include the development of unprovoked relapses, resulting in new damage to the CNS or worsening of existing neurological symptoms [2]. The course of MS is relatively stable between relapses, and this phase is referred to as the remission period [3]. The most important clinical marker of MS activity is the number of relapses [4].

Immunological changes associated with MS disease progression have been extensively studied. T cells play a central role in disease pathogenesis [5]. In recent years, B cells have also been shown to mediate MS pathogenesis. Evidences of B-cell activity include the observations of intrathecal immunoglobulin G production and B-cell expansion in MS lesions, as well as the therapeutic effects of plasma exchange and anti-CD20–based therapies [6]. Despite extensive research designed to identify specific MS biomarkers, no antibodies or immunological markers of MS have yet been validated for clinical use [7].

The major survival factor for B cells is B cell-activating factor (BAFF), a member of the tumour necrosis factor family. BAFF plays roles in the development of several autoimmune conditions and in the maintenance of inflammation [8]. Serum BAFF levels were found to be increased in some patients suffering from systemic lupus erythematosus, primary Sjögren’s syndrome, rheumatoid arthritis, immune thrombocytopenia, systemic sclerosis, myeloperoxidase-autoantibody associated renal vasculitis, myasthenia gravis, Graves’ disease, anti-glomerular basement membrane antibody disease, or MS [9–18].

Notably, BAFF has been demonstrated to accumulate in active demyelinating lesions of the human brain [18], and BAFF levels have been shown to be higher in the CSF during relapse in MS patients [19]. Increased BAFF levels had detrimental effects in a MS animal model, based on the observation that inhibition of BAFF reduced disease severity [8].

Over the last 20 years, numerous treatments designed to decrease MS relapse rates have become available [20], including therapies utilizing interferon (IFN)-β and glatiramer acetate (GA). The latter disease-modifying treatments (DMTs) are considered first-line treatment options [21]. However, only 3 relatively small studies have investigated the influence of IFN-based treatments on blood BAFF levels in MS. Although, Krumbholz *et al*. (2008) reported similar BAFF serum protein concentrations in untreated MS patients and HC, they showed that treatment with (IFN)-β leads to strong upregulation of BAFF in blood leucocytes and serum [22]. Hedegaard *et al*. (2011) prospectively recruited 26 MS patients in remission before initiation of IFN-β treatment and followed these patients for 26 months [23]. BAFF levels were elevated as early as 9–12 h postinjection and remained at levels above baseline during therapy without any increase in disease activity. Moreover, the increase in BAFF levels was accompanied by an 11–33% decrease (rather than the expected increase) in autoantibodies to myelin basic protein [23]. Finally, Vaknin-Dembinsky *et al*. (2010) studied 11 patients with MS, and found that treatment with IFN-β and GA was associated with higher serum BAFF levels. In this cross-sectional study, all samples were collected during remission, and BAFF levels were similar between untreated MS patients and healthy controls [24].

Treatment with the anti-CD52 monoclonal antibody alemtuzumab has been shown to increase BAFF levels. In a cross-sectional study of 78 patients with relapsing-remitting MS (RRMS), serum BAFF levels increased 3-fold during the first month after treatment and remained significantly elevated compared to baseline levels at all subsequent time points up to 12 months. Interestingly, serum levels of B-cell maturation antigen (BCMA), proliferation-inducing ligand (APRIL), and transmembrane activator (TACI) were unchanged after alemtuzumab treatment [25].

Breakthrough relapses are commonly treated with short courses (3–5 days) of high doses of intravenous methylprednisolone (IVMP). This short-term steroid treatment improves relapse symptoms but does not have a long-term effect on disease progression [26]. To date, the influence of a short course of IVMP on blood BAFF levels in MS patients has not been investigated. However, chronic oral (6–60 mg) steroid treatment of Wegener granulomatosis patients was shown to decrease BAFF concentrations to levels present in healthy individuals [27]. Increased BAFF levels in the CSF and serum were also reported after GA treatment in 11 MS patients. Untreated MS patients and healthy controls did not differ in their serum BAFF levels. All samples were collected during remission [24].

In the present prospective longitudinal study, we addressed the question of how blood BAFF levels are associated with the clinical course of RRMS, including relapses, and short-term steroid (IVMP) treatment or DMTs.

## Materials and Methods

### Patient and control groups

The study group consisted of 170 consecutively enrolled patients with RRMS, including 103 women and 67 men. The mean age of the study group was 39.2 ± 11.5 years, and the mean disease duration was 11.3 ± 9.1 years. All participants were recruited between February 2010 and October 2013 from the Multiple Sclerosis Centre at West-Tallinn Central Hospital (Estonia). The mean follow-up time of the study was 2.3 years. Patients with RRMS were recruited during a relapse or during remission. Only relapses with duration of up to 30 days were included. Patients were defined as being in a stable phase if they remained relapse-free for at least 3 months.

Patients with MS were evaluated at recruitment, on the first day during a visit for relapse, and certain time points after relapse (1–2 weeks, 1 month, 3 months, and 6 months after the first day during a visit). If a patient with a documented relapse during the study had further relapses, these events were registered as new relapses, and they were evaluated and followed-up accordingly. MS was diagnosed according to the McDonald criteria. Relapses were defined by patient-reported symptoms or objectively observed signs typical of an acute inflammatory demyelinating event in the CNS (current or previously established), with a duration of at least 24 hours and in the absence of fever or infection [26, 28].

Neurological disability was evaluated by the Expanded Disability Status Scale (EDSS) [29]. Whenever possible, the EDSS was evaluated at each visit by the same neurologist. Changes in the EDSS results were not required to confirm relapse diagnosis. Of the study participants, 63% were receiving DMTs. First-line treatment included IFN-β and GA. Mitoxantrone or cyclophosphamide was used as a second-line treatment, as needed. All relapses were treated with 1 g of IVMP for 5 consecutive days. Second-line treatments consisting of natalizumab and fingolimod were not available during the study; therefore, only the aforementioned treatments were used to treat MS patients.

The healthy control (HC) group included 49 reportedly healthy volunteers (40 women and 9 men), with a mean age of 37.2 ± 11.3 years. Another control group consisted of 38 patients (30 women and 8 men, mean age: 43.6 ± 10.8 years) with acute lower back pain (LBP) without reported autoimmune diseases, who had been referred to West-Tallinn Central Hospital for physiotherapy consultation.

Blood samples were obtained at baseline (stable phase), if available; during relapse before the first dose of IVMP was administered; and during follow-up visits at 2 weeks, 1 month, 3 months, and 6 months after a relapse. Informed consent was obtained from all participants.

### Ethics Statement

The study was approved by the Tallinn Medical Research Ethics Committee (Reg. no 1932, 21.01.2010) and written informed consent was obtained from all participants.

### Enzyme-linked immunosorbent assay (ELISA)

Venous blood was collected in EDTA tubes and centrifuged within 1 hour of collection. Plasma was carefully removed from the blood samples to avoid aspiration of thrombocytes. Plasma was stored at -80°C until testing and thawed only once. Plasma BAFF levels were measured with the Human BAFF/BLyS/TNFSF13B Quantikine ELISA kit (R&D Systems, Minneapolis, USA), according to the manufacturer’s protocol. The minimal detectable dose of BAFF in plasma was 2.6 pg/ml.

### Statistical analysis

The R 3.0.2 language and environment (Free Software Foundation, Boston, MA) [30] and GraphPad Prism 5 (GraphPad Software, La Jolla, CA) software programs were used for statistical analyses and figure preparation. The mean ± standard deviation is reported for descriptive statistics. BAFF measurements were log-transformed for statistical analyses to reduce departures from the normal distribution. Correlation between variables was performed by the Spearman rank correlation coefficient. The Fisher exact test was used to assess the relationship between 2 categorical variables. To compare proportions between two groups, a 2-tailed proportion test was used. The Welch two sample *t*-test, Fligner-Killeen test or Mann–Whitney *U*-test was used to compare characteristics between two groups. For more than 2 comparisons, one-way ANOVA or the Kruskal-Wallis test were used, followed by the Bonferroni correction or Tukey Honestly Significant Differences (Tukey HSD test) for multiple comparisons. For Bonferroni, multiple corrections for different numbers of hypotheses and *P-*values were used, as follows: *P*-values ≤ 0.0167 were considered significant for three hypotheses and *P*-values ≤ 0.0083 were considered significant for six hypotheses.

Multiple linear (lm) and logistic regression (glm) models were performed to test statistical significance. *P-*values < 0.05 were considered statistically significant. For evaluating the prediction accuracy of the logistic regression model, the area under the ROC-curve (AUC) was calculated (AUC of 0.5 indicates minimal and 1.0 maximal discrimination power).

## Results

### General characterization of the study groups

From the 170 recruited MS patients, 186 relapses were recorded during the entire study period (Table 1). There were 74 patients with 1 relapse, 31 patients with 2 relapses, 10 patients with 3 relapses, and 5 patients with 4 relapses. MS patients were divided into 3 subgroups on the basis of disease progression: 94 patients were recruited during a relapse (relapse RRMS [rRRMS] group), 50 patients were recruited during the stable phase and remained stable during the whole study period (stable RRMS [sRRMS] group), and 26 patients were recruited during a remission phase and relapsed during the follow-up period (remission-relapse RRMS [rrRRMS] group). All patients who experienced 3 or more relapses were classified into the rRRMS group.

**Table 1:** Patients with multiple sclerosis (MS) characteristics.

Characteristics	rRRMS	rrRRMS	sRRMS
	n = 94	n = 26	n = 50
	(M 38; F 56)	(M 9; F 17)	(M 20; F 30)
**Age, mean (SD), y**	40.2 (11.6)	40.7 (12.8)	36.5 (10.4)
**Disease duration, median (range), y**	10 (0–52)	10 (0.3–35)	7 (0.1–39)
**Disease duration groups (%)**			
≤ 1	4 (4.2)	2 (7.6)	7 (14.0)
1–10	45 (47.9)	12 (46.2)	29 (58.0)
>10	45 (47.9)	12 (46.2)	14 (28.0)
**Number of relapses during the study**	154	32	0
1 relapse	54	20	0
2 relapses	25	6	0
3 relapses	10	0	0
4 relapses	5	0	0
**Previous 3 years relapses (%)**			
Yes	73 (77.7)	21 (80.8)	29 (58.0)
No	21 (22.3)	5 (19.2)	21 (42.0)
**EDSS before study entry, median (range)**	3.0 (0–6.5)	3.5 (0–6.0)	2.0 (0–6.0)
**EDSS groups before study entry (%)**			
0–3.5	57 (60.6)	20 (76.9)	40 (80)
4–5.5	12 (12.8)	5 (19.2)	9 (18)
≥ 6	16 (17.0)	1 (3.9)	1 (2.0)
Not known	9 (9.6)	0 (0)	0 (0)
**EDSS during relapse, median (range)**	4.0 (0–8.0)	4.0 (0–6.0)	–
**Immunomodulatory treatment on the recruitment into the study (%)**			
Untreated	37 (39.4)	9 (34.6)	17 (34.0)
All receiving treatment	57 (60.6)	17 (65.4)	33 (66.0)
Interferon β^1^	18 (31.6)	9 (52.9)	23 (69.7)
Glatiramer acetate^2^	31 (54.4)	7 (41.2)	10 (30.3)
Immunosuppression treatment^3^	8 (14.0)	1 (5.9)	0 (0.0)
**Treatment changed (%)**			
Changed	15 (16.0)	7 (26.9)	2 (4.0)
Unchanged	79 (84.0)	19 (73.1)	48 (96)

^1^ Interferon β i.e Betaferon®, Rebif® or Avonex®

^2^ Glatiramer acetate i.e Copaxone®

^3^ Cytostatics includes mitoxantrone and cyclophosphamide

The mean ages of patients in the 3 MS subgroups were similar; however, the mean disease durations were different (Kruskal-Wallis rank sum test, *P* = 0.016). Patients in the rRRMS and rrRRMS groups had similar disease durations (Mann–Whitney *U*-test, *P* = 0.66). Patients in the sRRMS group had shorter disease durations compared to patients in the rRRMS group (Mann–Whitney *U*-test, *P* = 0.0048), but not compared to patients in the rrRRMS group (Mann–Whitney *U*-test, *P* = 0.08). Patients in the sRRMS group included more individuals with a disease history of up to 1 year and fewer individuals with prolonged presentation of disease (>10 years) compared to rRRMS participants (Fisher exact test, *P* = 0.0248; Table 1). Moreover, the follow-up period of the study differed for three MS subgroups (Kruskal-Wallis rank sum test, *P* = 0.00067). It was shorter for the rRRMS group compared to the sRRMS and rrRRMS groups (Mann–Whitney *U*-test, *P* = 0.00014 and *P* = 0.030, respectively). The follow-up period was not different for the sRRMS and rrRRMS groups ((Mann–Whitney *U*-test, *P* = 0.29). There were no statistically significant differences between gender distributions among the 3 RRMS groups.

The median EDSS scores before study entry were 3.0 (range: 0–6.5) for the rRRMS group, 3.5 (range: 0–6.0) for the rrRRMS group, and 2.0 (range: 0–6.0) for the sRRMS group. These differences were not statistically significant. The EDSS score before study entry was not available for nine patients. The median EDSS score during relapse was 4.0 in both relapsing groups (range: 0–8.0 for the rRRMS group and 0–6.0 for the rrRRMS group).

The number of relapse-free patients during the last 3 years before study entry was significantly different between MS subgroups (Fisher exact test, *P* = 0.0341). In the sRRMS group, 42% had no documented relapses during the previous 3 years. In the rrRRMS and rRRMS groups, only 19.2% and 22.3% of patients, respectively, were relapse-free (Table 1).

Analysis of the entire dataset demonstrated differences between the mean ages of participants in the HC, LBP, and MS groups (ANOVA, *P* = 0.0312). *Post hoc* analysis showed that participants in the LBP group were significantly older than participants in the HC group (mean age: 43.6 *vs*. 37.2 years; Tukey HSD test, *P* = 0.0026). There was a trend for participants in the HC group to be younger than patients in the MS groups (Tukey HSD test, *P* = 0.084).

### Plasma BAFF levels in the study groups

The median (interquartile range [IQR]) plasma BAFF level in patients with MS was 1041 pg/ml (884–1223 pg/ml), with plasma BAFF levels of 1007 pg/ml (832–1129 pg/ml) in the rRRMS group, 1007 pg/ml (960–1062 pg/ml) in the rrRRMS group, and 1211 pg/ml (974–1391 pg/ml) in the sRRMS group. In the control groups, the plasma BAFF levels were 1027 pg/ml (841–1187 pg/ml) in the LBP group and 850 pg/ml (759–972 pg/ml) in the HC group.

**Fig 1:**
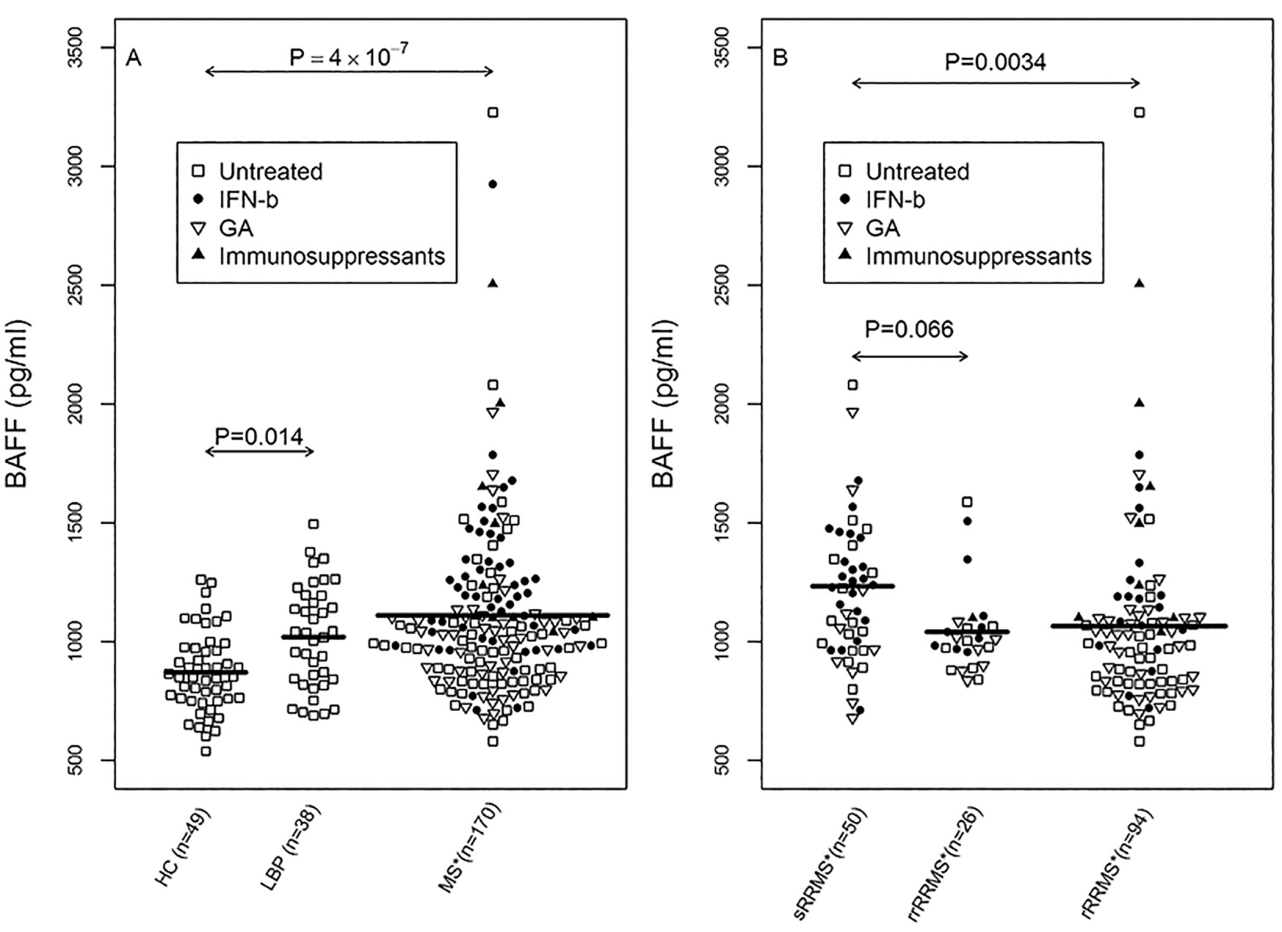
Average B cell-activating factor (BAFF) levels in the plasma of multiple sclerosis (MS) patients and controls. Bee Swarm plot of: (A) Plasma BAFF levels in healthy controls (HC group), patients with lower back pain (LBP group), and all MS patients. (B) Plasma BAFF levels in MS patient subgroups: stable MS patients (sRRMS), remission-relapsing MS patients (rrRRMS), and relapsing MS patients (rRRMS). Means and the results of Tukey HSD test are shown. Untreated (□) and IFN-β (●), GA (▽) or immunosuppressant (▲) treated individuals. *Average concentration of BAFF in patients during the follow-up period.

Average plasma levels of BAFF differed significantly during follow-up between groups (Fig 1A; ANOVA, *P* = 8.8 × 10^−7^). *Post hoc* analysis demonstrated significantly higher BAFF levels in patients with MS than in the HC group (Tukey HSD test, *P* = 4 ×10^−7^). BAFF levels also differed among MS subgroups (Fig 1B; ANOVA, *P* = 0.0039). Plasma BAFF levels were higher in the sRRMS group compared to levels in the rRRMS (Tukey HSD test, *P* = 0.0034). We found also a trend for higher BAFF levels in the sRRMS group compared to the rrRRMS group (Tukey HSD test, *P* = 0.066). However, mean plasma BAFF levels did not differ between the rRRMS and rrRRMS groups (Tukey HSD test, *P* = 0.99); a higher variance was detected in the rRRMS group compared with the rrRRMS group (Fligner-Killeen test, *P* = 0.0044). Since relapsing patients had similar clinical characteristics, we combined all patients with MS who experienced relapses during the study (rRRMS and rrRRMS groups) into a single combined relapsing group (crRRMS) for further statistical analysis. Of note, BAFF plasma levels were significantly lower in the crRRMS group compared to the sRRMS group (*t-*test, *P* = 0.0009). Plasma BAFF levels in the LBP group were elevated compared to levels in the HC group (Tukey HSD test, *P* = 0.014; Fig 1A). However, plasma BAFF levels were not associated with age or gender in the HC, LBP or MS group. In the MS group, BAFF level did not correlate with disease duration or EDSS (before study entry), nor was it associated with status of relapses during the 3-year period before enrolment in the study.

### Correlation between plasma BAFF levels and blood cells or inflammatory biomarkers

Analysis of the correlation between plasma BAFF levels and routinely used clinical blood biomarkers revealed weakly positive correlations with monocyte count (Spearman’s test, *P* = 0.0004, *R* = 0.1616) and basophil count (*P* = 0.0047, *R* = 0.1304). The average basophil counts were higher in crRRMS compared to sRRMS patients (median [range]: 0.01 (0–0.05) × 10^9^ and 0.02 (0–0.09) × 10^9^ cells/L, respectively; Mann–Whitney *U*-test, *P* = 0.0009). No correlation was observed between plasma BAFF levels and other blood cell types (*i*.*e*., neutrophils, lymphocytes, or eosinophils) or inflammatory markers (*i*.*e*., C-reactive protein or erythrocyte sedimentation rates).

### Characteristics of the DMTs in the RRMS subgroups

DMTs were used with similar frequency among all RRMS subgroups (60.6% in rRRMS, 65.4% in rrRRMS, and 66.0% in sRRMS). Nevertheless, a statistically significant difference was detected in the selection of drugs used between respective groups (Fisher exact test, *P* = 0.0162). For example, IFN-β treatment was more frequently used for participants in the sRRMS group compared to individuals in the crRRMS group (two-tailed proportion test, *P* = 0.003; Table 1). Immunosuppressants (mitoxantrone, cyclophosphamide) were used more frequently by participants in the rRRMS group compared to the rrRRMS group (14.0% *vs*. 5.9%, respectively), although that difference was not statistically significant due to the small number of patients treated with immunosuppressants.

### Correlation between plasma BAFF levels and DMTs in multiple sclerosis patients

**Fig 2:**
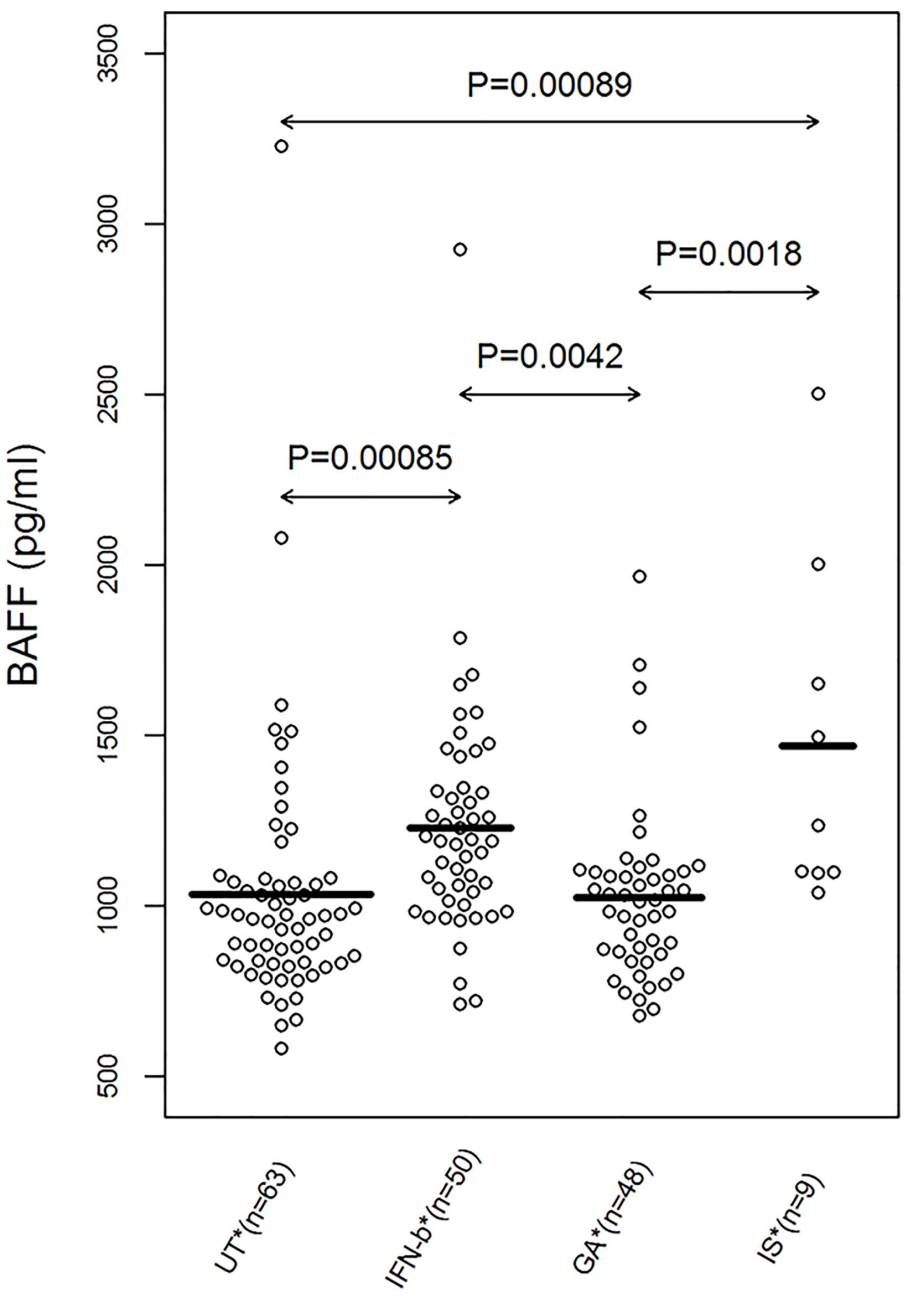
Average BAFF plasma levels and disease-modifying treatments (DMT). Bee Swarm plot of BAFF level in untreated (UT), IFN-β, glatiramer acetate (GA) and immunosuppressant (IS)-treated groups were shown. Means and the results of Tukey HSD test are shown. *Average BAFF values during treatment were used. In case of medication change, the new average BAFF was analysed separately.

There were statistically significant associations between plasma BAFF levels and the DMT used among MS patients (Fig 2; ANOVA, *P* = 4.6 × 10^−6^). BAFF levels were significantly higher in patients receiving IFN-β treatment compared to untreated MS patients (Tukey HSD test, *P* = 0.00085) and patients treated with GA (Tukey HSD test, *P* = 0.0042). Similarly, MS patients treated with immunosuppressants presented with higher average BAFF levels compared to untreated and GA treated MS patients (Tukey HSD test, *P* = 0.00089 and *P* = 0.0018, respectively). There were no significant differences in BAFF levels between MS patients on GA therapy and untreated MS patients, or between MS patients treated with IFN-β and those treated with immunosuppressants (Fig 2). However, untreated MS patients have statistically significantly higher BAFF level compared to HC (*t*-test, *P* = 0.0061). Stratification by treatment showed that BAFF level was statistically significantly higher in the untreated MS patients of the sRRMS group compared with the crRRMS subgroup (*t-*test, *P =* 0.0067, Figure A in S1 Appendix). Among the IFN-β treated patients, we demonstrated also a trend for higher BAFF in the sRRMS subgroup compared to crRRMS subgroup (*t-*test, *P =* 0.065, Figure Ba in S1 Appendix). Moreover, some of the initially stable patients relapsed during the study period and these cases were re-grouped from the sRRMS into the rrRRMS subgroup. However, we demonstrated that BAFF level tended to be relatively lower in the IFN-treated patients of the rrRRMS group compared with the stable IFN-β-treated patients (*t*-test, *P =* 0.0545, Figure Bb in S1 Appendix).

### Modelling the association between DMTs, BAFF levels, and clinical blood biomarkers in the context of multiple sclerosis disease presentation

To distinguish the confounding effect of the different factors, we conducted linear and logistic multiparametric regression analysis (glm and lm) to establish whether and how blood biomarkers (including BAFF levels), clinical characteristics and disease history were related to MS subgroups. We included the factors, associated with MS subgroups in previous statistical analysis, into the logistic regression models. The Model 1 (RRMS subgroup ~ BAFF level+DMT+EDSS+relapses during the previous 3 years +basophil count+age+gender) revealed that the crRRMS group had lower BAFF levels (*P* = 0.0022 adjusted for the other confounders), at least one relapse during previous 3 years (*P* = 0.012 adjusted for the other confounding variables) and had tendency for higher numbers of basophils in the peripheral blood (*P* = 0.06, adjusted for other confounders). Treatment (DMT) and disability (EDSS) were not related to the MS subgroups after adjusting (controlling) variables with the other confounding factors in the model. Replacement of EDSS with disease duration showed that longer disease duration and higher basophil account were also a statistically significant factors for grouping into crRRMS (respectively *P* = 0.023 and *P* = 0.039, adjusted for the other confounders). The prediction ability of BAFF level together with selected clinical data for the RRMS subgroups (Model 1) yielded an AUC of 0.83, and BAFF level alone without clinical data yielded an AUC of 0.67. Interestingly, adding basophile count to BAFF level data increased the AUC of the model up to 0.79 (Figure C in S1 Appendix). Collectively, analysis of Model 1 showed that two investigated biomarkers (BAFF level and basophile account) and two clinical characteristics (disease duration and relapses during the previous 3 years), but not DTM or disability level (EDSS), were independently related to the status of the RRMS subgroup.

In the next analysis (Model 2), we investigated which disease characteristics were associated with plasma BAFF levels. This linear regression model (BAFF level~ RRMS subgroup+ DMT+EDSS+ disease duration+ relapses during the previous 3 years +basophil count+age+gender) analysed the level of BAFF as a dependent variable and the RRMS subgroup, DMT, EDSS, basophil counts, disease duration, relapses during the previous 3 years and patients’ age or gender as independent variables. The model demonstrated that plasma BAFF levels were higher in MS patients treated with IFN-β or immunosuppressants (*P* = 0.00037 and *P* = 0.0007, respectively, adjusted for the other confounding factors). Interestingly, treatment with GA did not appear to affect BAFF levels. Irrespective of treatment, the RRMS subgroup was also associated with BAFF level (*P* = 0.0016, adjusted for the other confounders). However, several other cofactors in the model (basophile counts, age, gender, EDSS, disease duration and relapses during the previous 3 years) were not associated with BAFF level.

Summarizing the results, we found that BAFF level was independently associated with two factors: treatment with IFN-β or immunosuppressants, and RRMS subgrouping. The latter was associated with disease duration, relapses during the last 3 years and basophil count in blood. Intriguingly, disability status was associated neither with MS subgroups nor BAFF level.

### Changes in plasma BAFF levels during MS relapses and the effect of IVMP treatment

**Fig 3:**
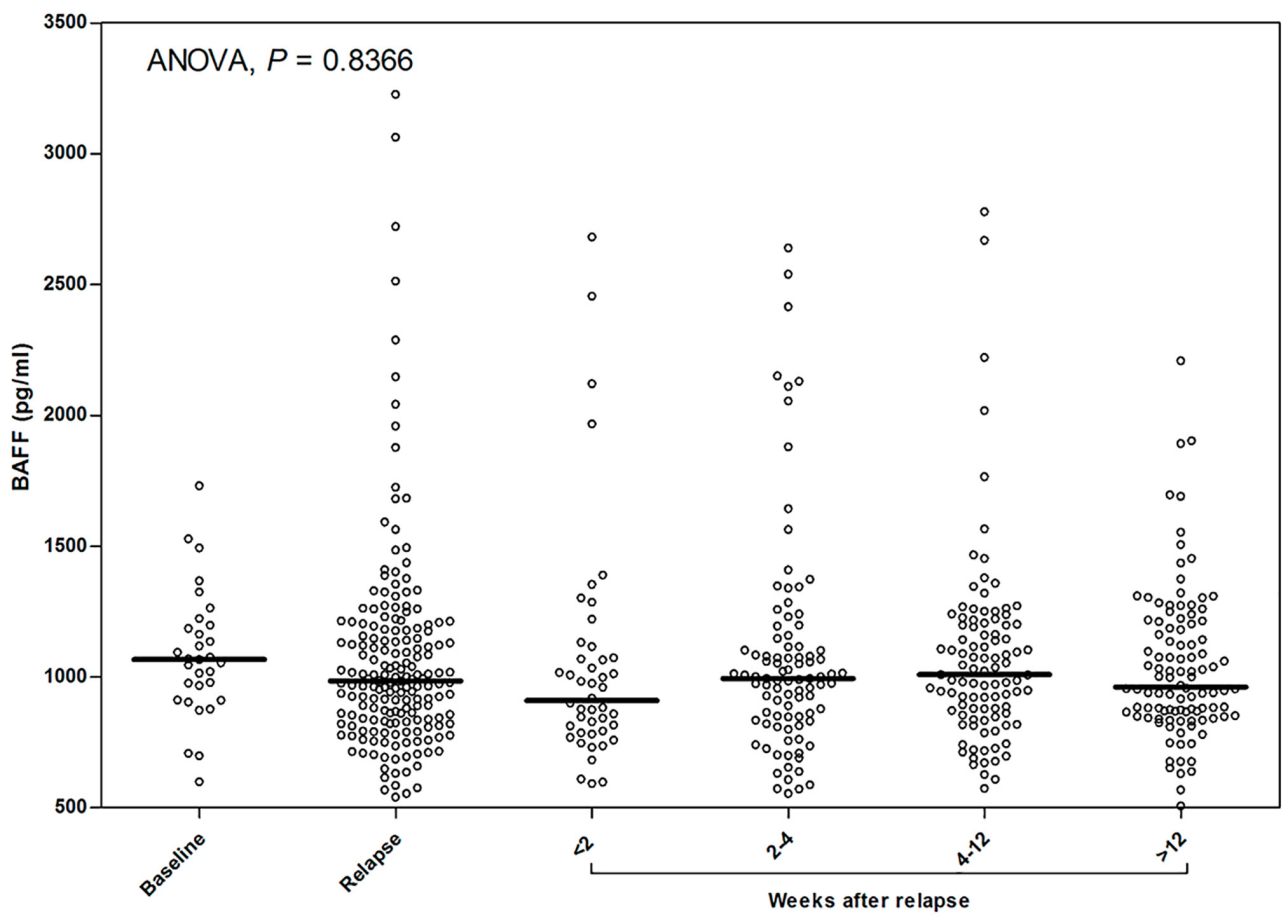
Changes in plasma BAFF levels during follow-up visits in the MS group. Bee Swarm plot and means of BAFF levels at baseline (n = 31), during relapse (n = 186) and 1–2 weeks (n = 46), 2–4 weeks (n = 93), 4–12 weeks (n = 99) or 12–24 weeks (n = 104) after the relapse were presented.

**Fig 4:**
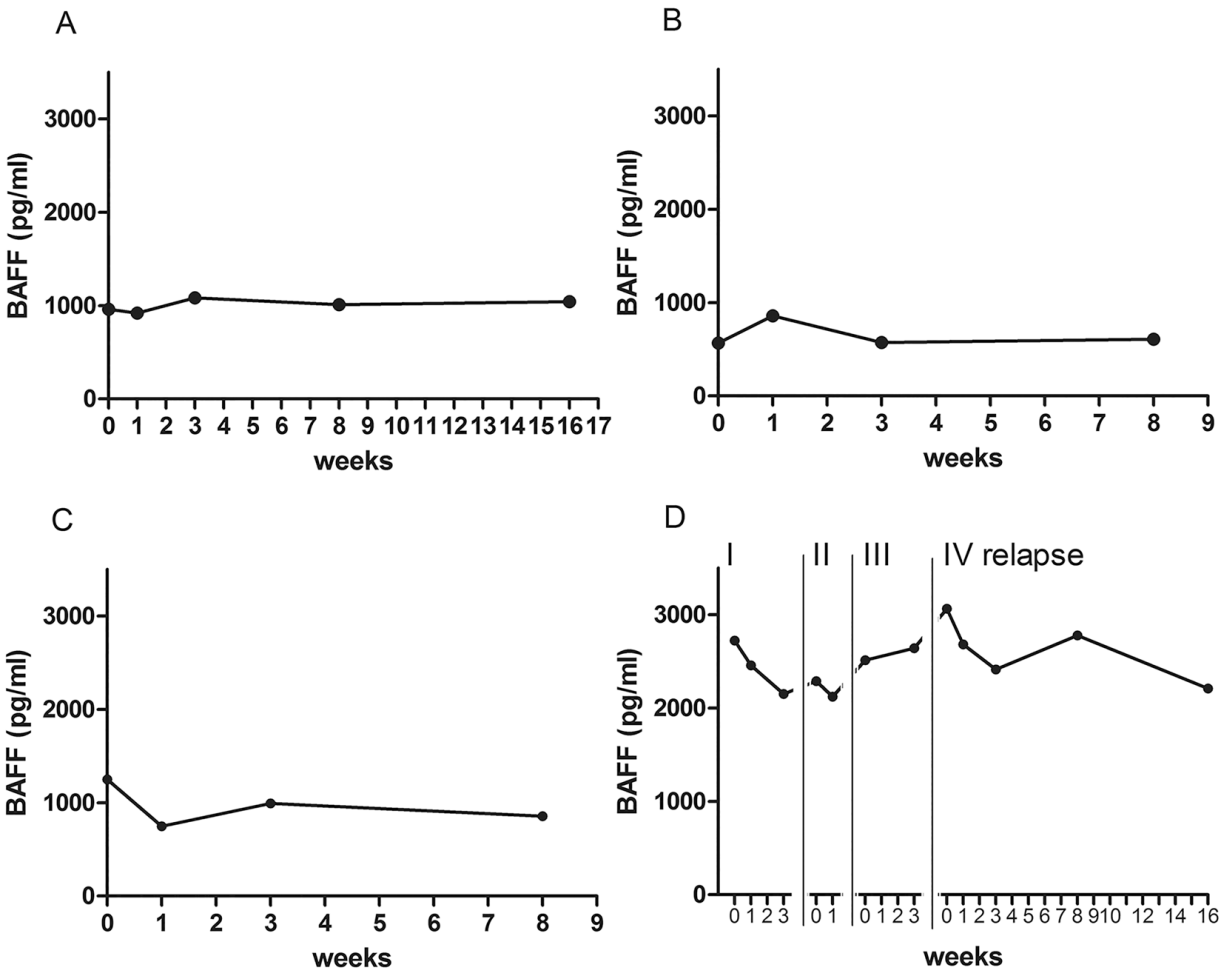
Examples of different patterns of plasma BAFF level changes during acute relapse in four patients with RRMS. (A) Patient with a uniform BAFF level pattern. (B) Patient with increased BAFF levels before steroid treatment. (C) Patient with decreased BAFF levels during acute relapse (before steroid treatment). (D) Patient with different patterns of BAFF levels during different relapse episodes.

Regrettably, not all dynamic points were available for all relapses (Fig 3). However, we did not observe statistically significant changes in mean plasma BAFF levels at the follow-up visits. In 65% (30/46) of relapses, the plasma BAFF levels before relapse, during acute relapse (before steroid treatment), and 2 weeks after relapse and IVMP treatment were similar. Only in 35% (16/46) of relapses did BAFF levels change by more than 20%. Seven patients had an increase and nine patients had a decrease in BAFF plasma levels within 2 weeks after relapse and treatment. Individual patients demonstrated variable patterns of change during relapses (Fig 4A–4C), and BAFF plasma levels in the same patient varied between multiple relapses (Fig 4D).

## Discussion

In the present prospective and longitudinal study, we determined how blood BAFF levels are influenced by the clinical course of MS, while utilizing an extensive collection of well-defined clinical and laboratory data. All MS participants belonged to a cohort of patients who had a documented history of MS and were consecutively enrolled at a national multiple sclerosis centre. During this 2.3-year study, we collected unique clinical material during the stable phase and during 186 relapses documented among 170 individual patients, allowing for analysis of data collected before and after IVMP treatment. To our knowledge, the size of this MS cohort is among one of the largest reported to date.

MS has traditionally been considered a T cell-mediated disease, and studies examining the importance of B cell-mediated immunity in disease progression have only recently been conducted [31]. Several studies using MS animal models have suggested that BAFF is critical for B-cell survival, and that an increase in serum BAFF level leads to an expansion of the B-cell compartment and autoimmunity in mice [8, 32]. According to the literature, the level of BAFF in serum or plasma is comparable [33]. However, to date, human studies have failed to establish a link between BAFF levels in serum or plasma, and disease severity or progression. BAFF levels in the CSF were linked to disease severity in humans [19, 34, 35], but a link between peripheral blood BAFF levels and disease severity could not be established [24, 36].

In the present study, peripheral blood BAFF levels were significantly higher in MS patients compared to levels in the HC group. Somewhat surprisingly, the highest BAFF levels were observed in stable MS patients, whereas BAFF levels were significantly lower in relapsing patients.

To our knowledge, this is the first study to demonstrate that higher blood BAFF levels are associated with a more stable course of MS. Stable patients (sRRMS group) who did not experience any relapses during follow-up had significantly fewer relapses before study entry (42% relapse-free patients in the sRRMS group *vs*. 22% in the relapsing group). On the other hand, relapsing patients recruited during a relapse (rRRMS) or who had a relapse during follow-up (rrRRMS) possessed surprisingly similar clinical profiles (mean age, disease duration, median EDSS scores during relapse, and relapse frequency before the study).

Unexpectedly, blood BAFF levels were also high in LBP patients and did not differ significantly from levels in the MS group. To our knowledge, blood BAFF levels in patients with LBP have not been studied previously. However, it was recently shown that B cells are recruited to the CNS in different neuroinflammatory diseases [37]. Fühlhuber *et al*. (2009) reported elevated BAFF in sera of patients with meningitis, encephalitis and neuroborreliosis [38]. Moreover, Hamzaoui *et al*. (2008) found elevated BAFF in Behçet's disease with CNS manifestation and Franciotta *et al*. (2011) reported correlation between BAFF and EBV-specific antibody levels [39, 40]. Therefore, the clinical importance of our finding should be addressed in future studies. The data generated during the current study are insufficient to explain this finding.

We also confirmed that IFN-β treatment is associated with a significant elevation of BAFF levels in blood, as reported previously [22, 23]. Moreover, BAFF levels changed relatively quickly (*i*.*e*., 9–12 h after the first dose of IFN-β) [23]. The increase in BAFF levels did not result in a more severe course of MS. In that respect, these results challenge the hypothesis that elevated blood BAFF levels are always associated with a more severe form of disease. Moreover, we demonstrated that the IFN-treated patients who entered the study in a stable phase of the disease but relapsed during the study had relatively low BAFF level compared with the patients who remained stable. Thus, attributing a negative effect to BAFF in the context of MS pathogenesis under all conditions may be too simplistic. This possibility is highlighted by a failed clinical trial (ATAMS study) that used Atacicept for neutralizing BAFF/APRIL and resulted in an unexpected increase in inflammatory activity [41]. A Phase II trial investigating a different BAFF blocker was terminated in 2011. No additional information about this latter trial is available regarding its effectiveness or the presentation of adverse effects that were associated with drug administration [42].

Other treatments with proven positive clinical effects (*e*.*g*., alemtuzumab and GA) have also been shown to induce higher BAFF levels in MS patients [24, 25]. However, serum levels of BCMA, APRIL, and TACI were unchanged after alemtuzumab treatment [25]. In the present study, we observed elevated blood BAFF levels in mitoxantrone-treated MS patients. To our knowledge, this is the first report describing an association between BAFF levels and the use of immunosuppressants in MS treatment. However, we observed similar blood BAFF levels in patients with or without GA. Because previously published and current studies of GA treatment have not been longitudinal and follow-up in nature, no definitive proof exists for a causal association between changes in blood BAFF levels and this type of treatment. These results support the idea that effective treatment of MS is associated with increased BAFF blood levels, and it could be argued that different DMTs may affect B cell-mediated immune responses differently.

The next issue addressed by the present study was to determine changes in blood BAFF levels in individual patients longitudinally. Surprisingly, blood BAFF levels remained stable for most MS patients (65%) during the follow-up period. In other words, the blood levels of BAFF were not influenced by relapse or steroid treatment. One third of MS patients presented with variable BAFF levels during relapse and after steroid treatment, suggesting that IVMP treatment can induce short-term changes in blood BAFF levels in some individuals. However, the time period of these changes may have been too small to detect changes after 2 weeks. It is possible that BAFF changes are very short-term, last a few hours after treatment and disappear within a week. The existence of such a possibility and its significance should be investigated further studies.

A statistically significant correlation was observed between BAFF levels and the monocyte and basophil counts. Activated myeloid lineage cells, including monocytes and basophils, have been described as primary producers of BAFF after activation with cytokines or microbial products [43–46]. Interestingly, the average basophil counts were higher in crRRMS compared to sRRMS participants. These findings suggest that basophils and monocytes could play a role in mediating MS pathogenesis. However, the present study was not designed to answer this question. Future studies are needed to clarify the role between innate and humoral immunity and their effects on MS *via* effects conferred by BAFF.

The major limitation of this study was the unequal number of collected plasma samples between groups. Relapsing patients were studied up to 4 times during the follow-ups after relapse, whereas stable patients and controls were studied only once at baseline. Because we did not have sera for patients before DMT, we could not directly assess the influence of the start of treatment on individual patients. In addition, the schedule of plasma collection was not ideal for capturing changes in blood BAFF levels during steroid treatment (*i*.*e*., 2 weeks could be too long for identifying changes that occur rapidly after treatment). Unfortunately, we were unable to assess BAFF concentrations in the CSF or to quantify B-cell subpopulations. Therefore, we could not determine the associations of BAFF levels in blood and CSF, or how B cells responded to higher blood BAFF levels. In summary, we confirmed that BAFF levels are increased in the peripheral blood of MS patients. The highest BAFF levels reflected a stable course in MS patients. It should be noted that among available effective DMTs, IFN-β and immunosuppressants result in an elevation of BAFF levels. Taken together, these findings raise additional questions regarding the role of BAFF in mediating the pathogenesis of MS.

## Supporting Information

S1 Appendix Supplementary Figures. (DOCX)
