# Supplementary material for: Changes in Blood B Cell-Activating Factor (BAFF) Levels in Multiple Sclerosis: A Sign of Treatment Outcome
Source: PLoS One. 2015 Nov 23;10(11):e0143393. doi: 10.1371/journal.pone.0143393 (PMC4658115; doi:10.1371/journal.pone.0143393)
Supplement: S1 Appendix — (DOCX) [file pone.0143393.s001.docx]

**S1 Appendix. Supplementary Figures.**

**
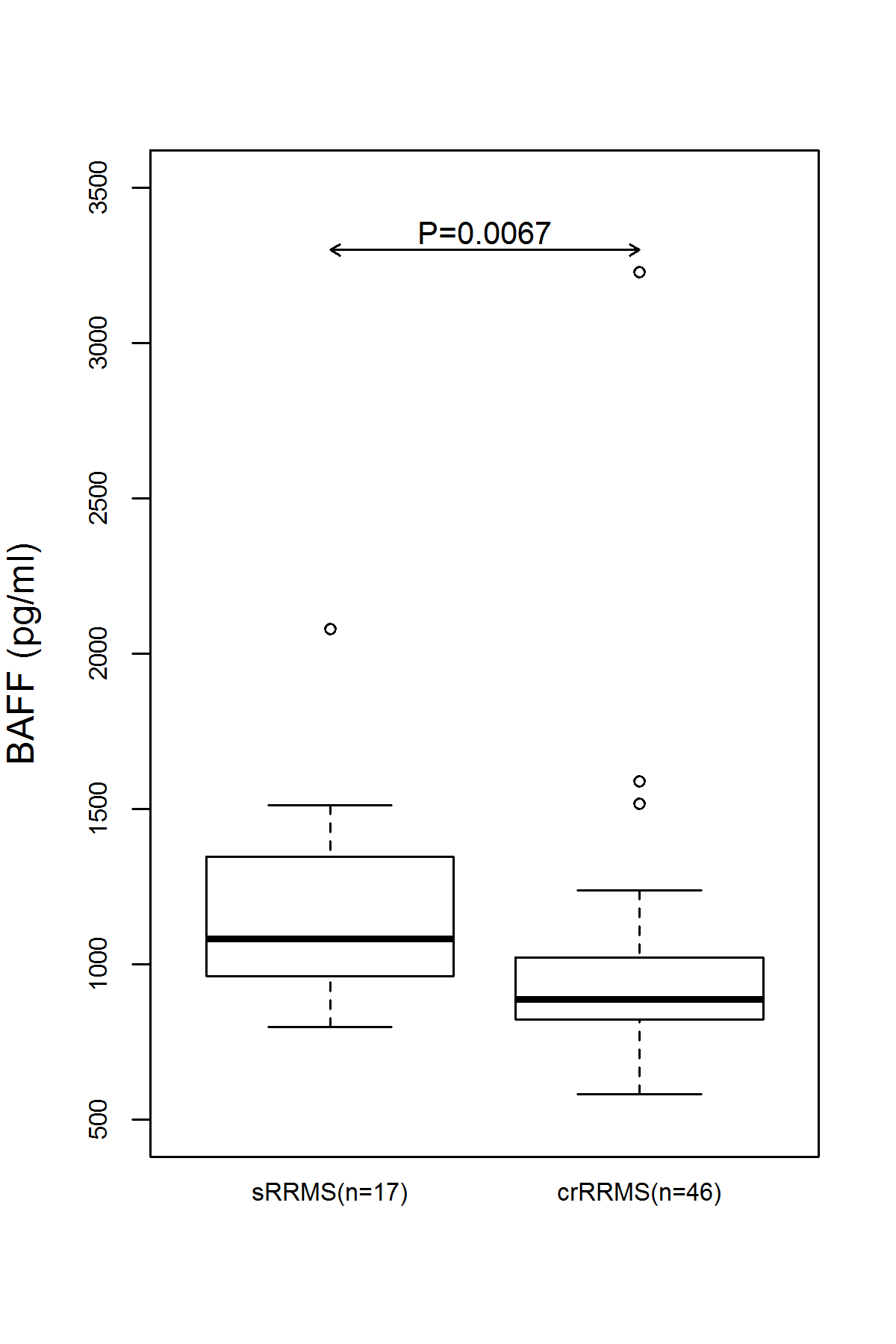
**

**Figure A. Average BAFF plasma levels of MS patients not treated with DMT.**

Boxplot of the BAFF level of untreated patients in the sRRMS group and crRRMS groups were shown. Stable patients demonstrated higher BAFF level compared to the relapsing patients (*t*-test, *P =* 0.0067).

**a b**


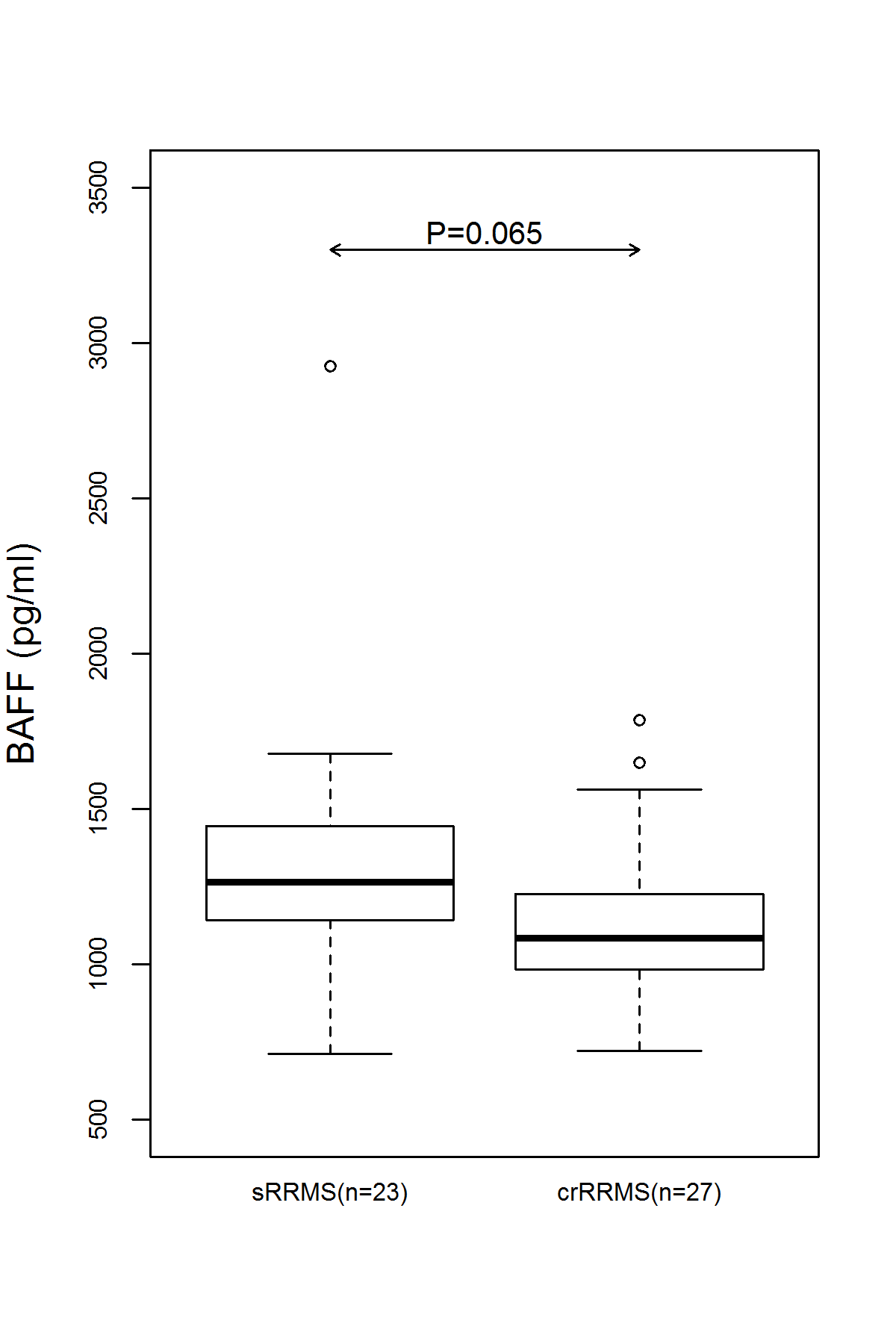

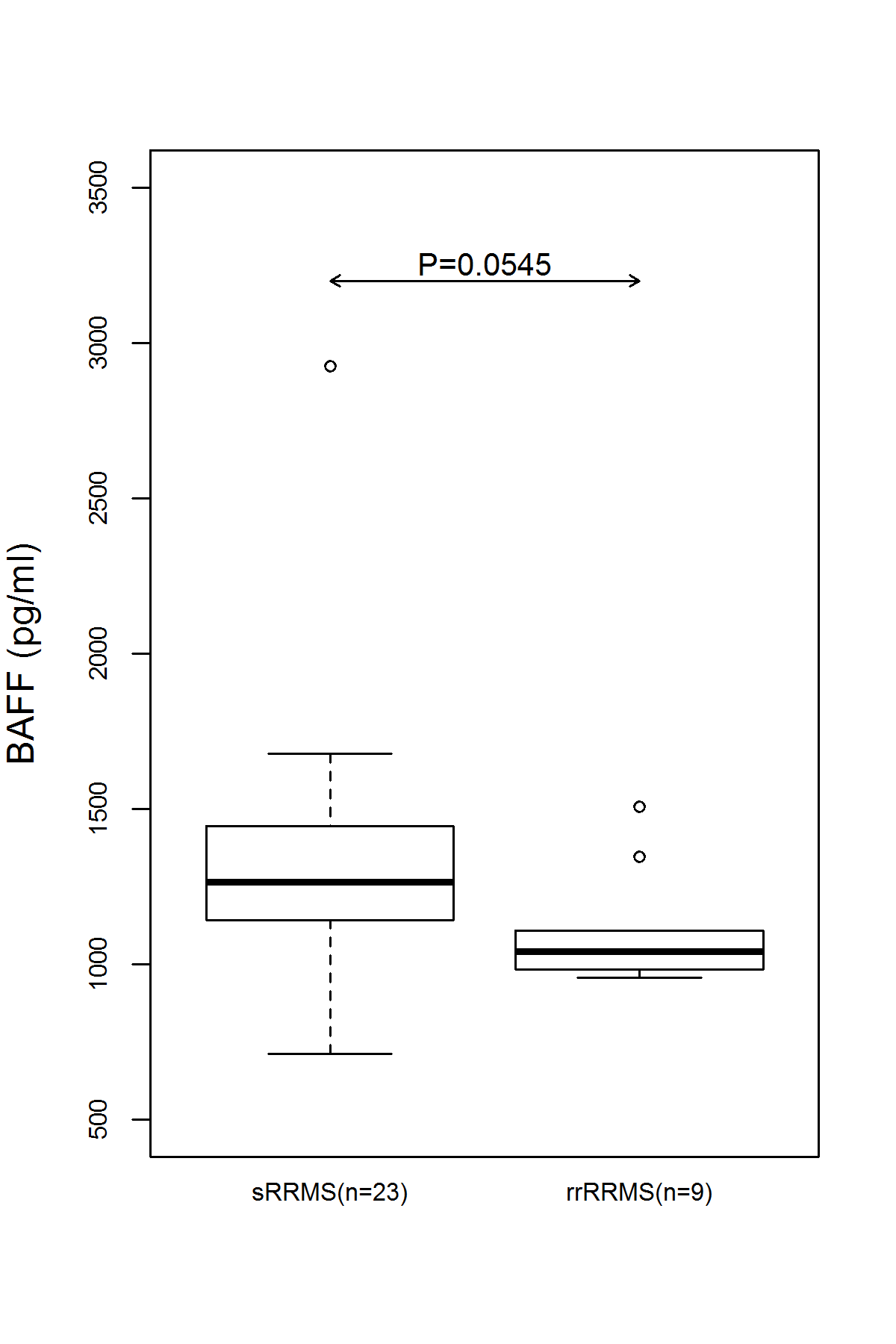


**Figure B. Average BAFF plasma levels of IFN-β-treated MS patients.**

Boxplot of BAFF level of: (a) IFN-treated patients in the sRRMS group and crRRMS groups. Stable patients had trend for higher BAFF level compared to the relapsing patients (*t*-test, *P =* 0.067); (b) IFN-β treated patients of the sRRMS group and rrRRMS. Stable patients had trend for higher BAFF level compared to the patients relapsing during the study (*t*-test, *P =* 0.0545).


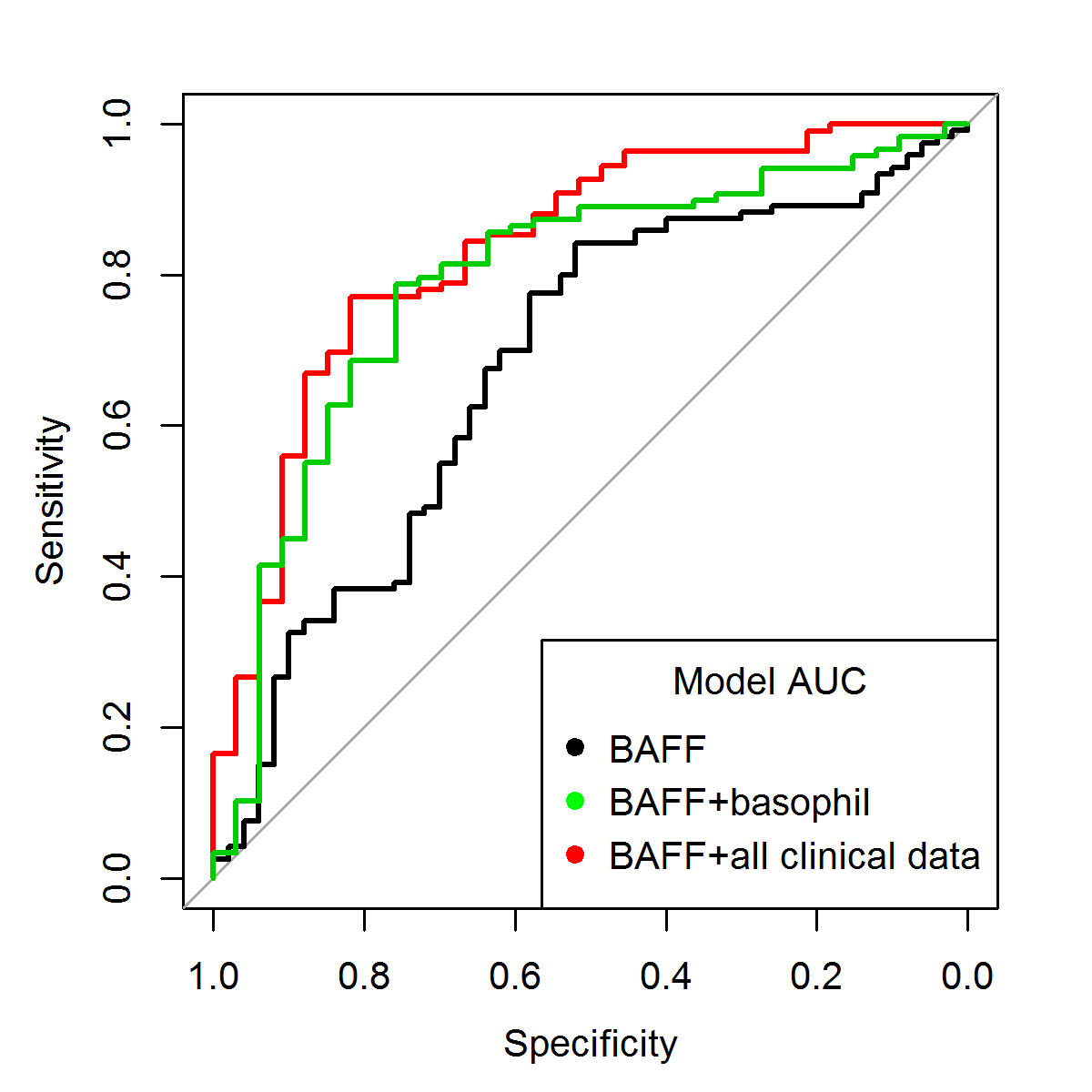


**Figure C. ROC curves of different models for discriminating the stable and relapsing MS subgroups (sRRMS/crRRMS).**

The area under the curve (AUC) was 0.83 for best-fit model (RRMS subgroup ~ BAFF level+ DMT+EDSS+relapses during the previous 3 years+basophil count+age+gender) (red), 0.79 for BAFF level+basophil count) (green), and 0.67 for BAFF level only (black).
